# The stylomastoid artery as an anatomical landmark to the facial nerve during parotid surgery: a clinico-anatomic study

**DOI:** 10.1186/1477-7819-7-71

**Published:** 2009-09-28

**Authors:** Tahwinder Upile, Waseem Jerjes, Seyed Ahmad Reza Nouraei, Sandeep U Singh, Panagiotis Kafas, Ann Sandison, Holger Sudhoff, Colin Hopper

**Affiliations:** 1 Department of Otolaryngology/Head and Neck Surgery, Charing Cross Hospital, London, UK; 2 The Ear Institute, University College London, London, UK; 3 UCLH Head & Neck Centre, London, UK; 4 Department of Surgery, University College London Medical School, London, UK; 5 Unit of Oral & Maxillofacial Surgery, Division of Maxillofacial, Diagnostic, Medical and Surgical Sciences, UCL Eastman Dental Institute, London, UK; 6 Department of Oral Surgery and Radiology, School of Dentistry, Aristotle University, Greece; 7 Department of Pathology, Imperial College & Charing Cross Hospital, London, UK; 8 Department of Otolaryngology, Head and Neck Surgery, Bielefeld Academic Teaching Hospital, Bielefeld, Germany

## Abstract

**Background:**

The identification of the facial nerve can be difficult in a bloody operative field or by an incision that limits exposure; hence anatomical landmarks and adequate operative exposure can aid such identification and preservation.

In this clinico-anatomic study, we examined the stylomastoid artery (SMA) and its relation to the facial nerve trunk; the origin of the artery was identified on cadavers and its nature was confirmed histologically.

**Methods:**

The clinical component of the study included prospective reviewing of 100 consecutive routine parotidectomies; while, the anatomical component of the study involved dissecting 50 cadaveric hemifaces.

**Results:**

We could consistently identify a supplying vessel, stylomastoid artery, which tends to vary less in position than the facial nerve. Following this vessel, a few millimetres inferiorly and medially, we have gone on to identify the facial nerve trunk, which it supplies, with relative ease. The origin of the stylomastoid artery, in our study, was either from the occipital artery or the posterior auricular artery.

**Conclusion:**

This anatomical aid, the stylomastoid artery, when supplemented by the other more commonly known anatomical landmarks and intra-operative facial nerve monitoring further reduces the risk of iatrogenic facial nerve damage and operative time.

## Background

Parotid surgery can at best be tricky and at worst ruinous. The crux of the matter remains the functional preservation of facial nerve (FN), through proper identification and preservation of the nerve at surgery [1].

The identification of the nerve can be difficult in a bloody operative field or by an incision that limits exposure; hence the use of anatomical landmarks, careful dissection with a 'bloodless field' and adequate operative exposure can aid such identification and preservation (Table 1). There are many anatomical variations in the position of the facial nerve. Since on an embryological basis the facial nerve grows into the developing parotid gland and is subject to many different anatomical variations that are not paralleled in branchial artery development. Teleologically this is an extension of evolutionary principles whereby a reliable blood supply is required and assured for other structures to develop around them. Hence one may argue that arteries or supplying vessels tend to vary in position less than nerves [2].

**Table 1:** Summary of the most common methods in identifying the facial nerve trunk.

**Surface landmarks**	**Intra-operative landmarks**	**Using instruments**
• Temporomandibular joint	• Retromandibular vein	• Use nerve stimulator
• Mastoid process	• Tragal pointer	• Use of nerve monitor
• Angle of the mandible	• Tympanomastoid fissure	
• Transverse process of the axis	• Styloid process	
	• Posterior belly of the Digastric	
	• Retograde dissection of a Peripheral branch to the main trunk	
	• Temporoparotid facia	
	• Extension of dissection from the vertical portion of the facial nerve within the mastoid (diagastric ridge)	

n the ctn guageasoconstrictordentified we rarely use diathermy

Approaches to the parotid gland need therefore to provide excellent exposure to allow unencumbered identification and dissection of the facial nerve, and complete excision of the pathological lesion. The standard approach is via a cervico-mastoid-facial incision, which provides good exposure and is relatively easy to perform [3].

In this clinico-anatomic study, we looked at the stylomastoid artery (SMA) and its relation to the facial nerve trunk; the origin of the artery was identified in cadavers and its arterial nature was confirmed histologically. The artery traverses the stylomastoid foramen with the facial nerve.

## Methods

The study protocol was approved by the local committee of the ethics for human research.

The clinical component of the study included prospective observation of 100 consecutive routine parotidectomies, noting the presence and variations of the stylomastoid artery. Where the surgical approach permitted in 56 of those cases (i.e. the posterior belly of the digastric muscle was dissected), the origin of the stylomastoid artery was also identified.

An information sheet explaining the aim of our study in simple non-scientific terms was given to each patient who was then asked to sign a consent form.

The anatomical component of the study involved dissecting 50 cadaveric hemifaces to assess the surgical anatomy of the stylomastoid artery; furthermore, the arterial nature of the vessel was confirmed by histologically.

Intraoperative clinico-anatomical data included: origin of the stylomastoid artery (SMA), recording the usual position and any variation of the facial nerve (FN) and assessing whether the stylomastoid artery was helpful in identifying the FN (Table 2).

**Table 2:** Intraoperative clinico-anatomical data.

**Study**	**Identified**	**Origin of SMA OA:PA**	**Usual position of FN**	**Variable position of FN**	**Was the SMA helpful?**	**Reasons for the SMA being unhelpful**
Clinical	80 cases	46:10	70%	30%	72/80	Bloody field
Cadaveric	50 cases	42:8	76%	24%	46/50	Poor tissue anatomy

**SMA**: stylomastoid artery; **FN**: facial nerve; **OA**: occipital artery; **PA**: posterior auricular artery.

A standard cervico-mastoid-facial incision was employed in both the clinical and anatomical components of the study.

### Description of the surgical access to the stylomastoid artery (traditional cervico-mastoid-facial approach)

The consented patient is placed under general anaesthesia (with hypotension if indicated) and positioned in a "Reverse Trendelenberg 30°" with the head turned to the opposite side.

The patient was draped in such a way so that the ear, corner of the eye, corner of mouth and neck are exposed. We use an 'opsite^®^' see-through adhesive plastic drape over the exposed areas and infiltrate the marked skin incision with a dilute tumescent vasoconstrictor solution.

Bipolar cautery and a working facial nerve stimulator were used (set at low mA) as part of routine practice. An assistant provided intelligent counter traction, whilst being aware of pressure induced nerve ischemia.

A cervico-mastoid-facial incision is made with a number 10 blade; the incision follows the anterior contour of the ear then curves gently behind the auricle, and extends anteriorly at least two of the "patient's fingerbreadths" beneath the lower border of the mandible. The parotid flap is raised with sharp iris scissors, with the blades spread at right angles to the capsule. Dissection continues to the anterior border of the gland (where the fascia overlying the masseter is visible). Posteriorly and inferiorly the gland is separated from the sternomastoid muscle. One should try and preserve at least the posterior division of the greater auricular nerve if oncologically justified. The parotid is separated from the cartilaginous portion of the external ear by careful scissor dissection until the tragal pointer is exhibited.

Haemostasis is achieved using a combination of head up position, vasoconstrictor solution, fine mosquito clips, small gauge ties and bipolar cautery. The posterior belly of the digastric muscle is separated from the gland and followed superiorly. The mastoid process is also identified. Working on a broad front, from below up and behind forwards the remaining parotid gland is separated from the cartilaginous ear, posterior belly of the digastric and mastoid tip.

Curved mosquito clips were used to elevate and separate the tissues before possible division. We often use surgical magnification to find the small arterial branch which usually overlies the main trunk of the nerve. The stylomastoid artery (SMA) is routinely used as a surgical landmark to identify the FN; this arterial branch may be divided later if necessary. Depending upon the location of the lesion, we work across a broad front tracing the facial nerve branches to the borders of the specimen. The wound is irrigated and the patient placed in a head down position and an anaesthetic Valsalva manoeuvre is carried out. Judicious haemostasis with cautery and ties is carried out before a large 'Haemovac' drain is carefully placed and the incision closed in layers with absorbable suture to deep tissues.

Cadaveric dissection was performed via a cervico-mastoid-facial incision and standard dissection was performed as described above. Clinical and gross anatomical photography was obtained from a Fujifilm Finepix 2 Megapixel Digital Camera, all surgical instruments were obtained from the Downs surgical^® ^catalogue.

## Results

**Figure 1:**
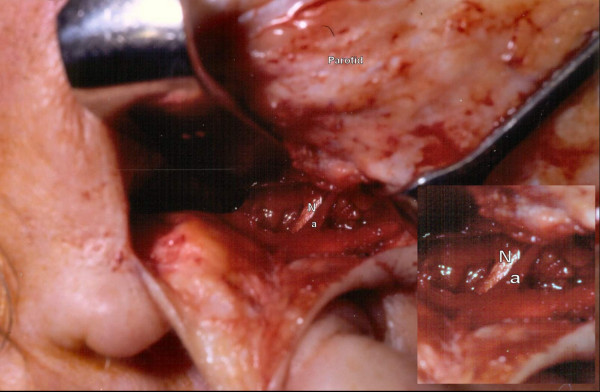
**Intraoperative dissection: showing stylomastoid artery located just above and superior to the facial nerve**. Inset shows a magnified view. N: nerve, a: sytlomastoid artery.

After prospective review of a 100 parotidectomies, we can consistently identify a supplying vessel, the stylomastoid artery. Following this vessel, a few millimetres inferiorly and medially, we have gone on to identify the facial nerve trunk, which it supplies, with relative ease (Figure 1). This has potentially shortened the overall mean duration of the operation however this is not a controlled primary outcome measure and is purely anecdotal. We would wish to advocate careful and timely dissection rather than 'rushed' surgery that may jeopardise patient outcomes.

After further cadaveric dissections (50 hemifaces), we determined this to be the SMA which is known to supply the facial nerve whilst accompanying it through the stylomastoid foramen. The arterial nature of the vessel was confirmed histologically.

**Figure 2:**
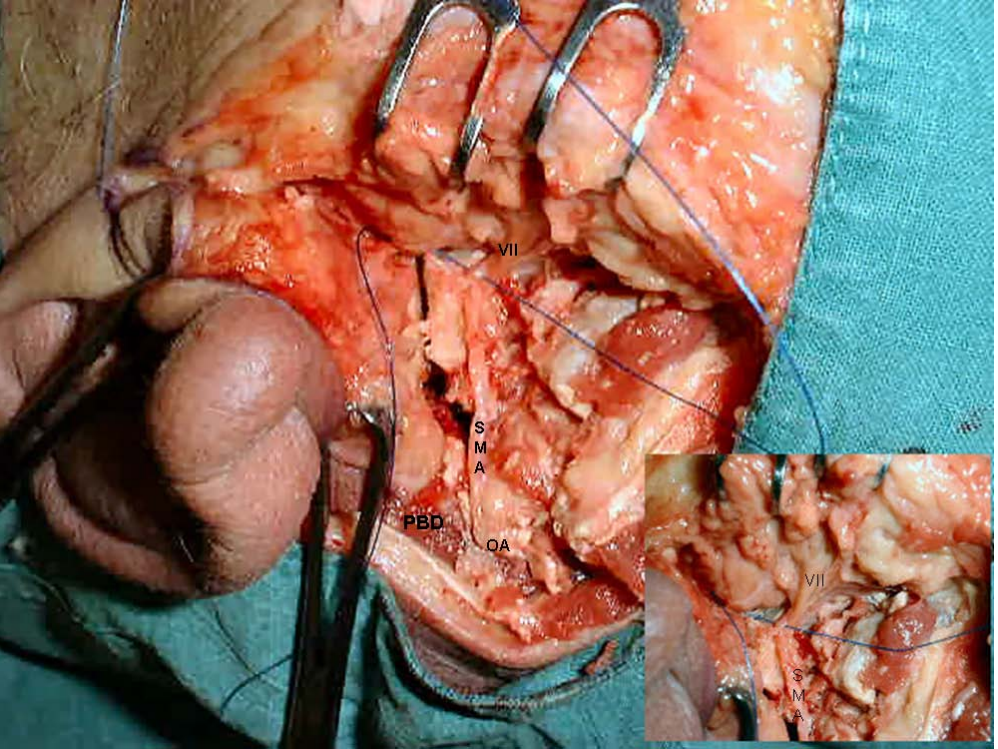
**Cadaveric dissection: displaying the stylomastoid artery (SMA) and underlying nerve (VII) located inferiomedially**. Inset shows magnified view with nylon between nerve and artery. The Mastoid process has been detached and sternomastoid muscle reflected inferiorly, with the cut posterior belly of the digastric muscle (PBD) displayed. Showing this vessel to be a branch of the occipital artery (OA).

**Figure 3:**
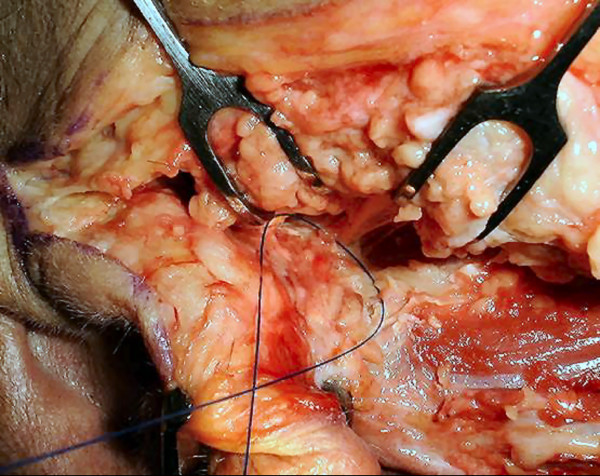
**Cadaveric dissection: showing the stylomastoid artery above the nerve with nylon between; this dissection revealed the artery to be a branch of the posterior auricular artery**.

In 80 of the 100 clinical cases, the SMA was clearly identified but in only 56 cases was it possible to determine the origin of stylomastoid artery from either the occipital artery or the posterior auricular artery during the dissection. In 8 cases the SMA was unhelpful because of previous soiling of the operative field with blood. The initial dissection technique must be meticulous and gentle to avoid this problem. In the cadaveric study, the post mortem changes in 4 specimens precluded location of the nerve just by using this technique (Table 2). The origin of the SMA is from one of the posterior branches of the external carotid artery that travel along the medial border of the posterior belly of the digastric muscle (Figure 2). We believe the vessel to be a minor branch of the occipital artery (in over three quarters of patients) and less frequently a branch of the posterior auricular artery (in nearly one quarter of patients) (Figure 3). The existence of this artery should now be common surgical knowledge.

## Discussion

In order to have safe and effective parotid gland surgery, knowledge of the anatomical landmarks to the facial nerve is essential. A wide range of landmarks have been reported in the literature. However, the reliability of these landmarks is still one of the main concerns since there is no conclusive evidence that any one landmark is better than the rest [4-8].

Pereira et al. [5] suggested that external palpable landmarks can be used to identify the facial nerve trunk quickly and safely. In a study that involved 40 human cadavers, they proposed that a centre of a triangle formed by the temporomandibular joint, the mastoid process and the angle of the mandible allowed a fast and safe identification of the facial nerve and may be of significant help during surgery around the parotid region. Pather and Osman [1] evaluated the relation of the surrounding anatomical structures and surgical landmarks to the facial nerve trunk through a micro-dissection on 40 adult cadavers. Their results showed that the posterior belly of digastric, tragal pointer and transverse process of the axis are consistent landmarks to the facial nerve trunk.

El-Hakim et al. [4] assessed the accuracy of using surrogate anatomic structures radiologically to predict the relation of parotid lesions to the intraparotid facial nerve. The retromandibular vein was identified as being the most accurate structure. Witt et al. [6] carried out a prospective study of 14 cadaver specimens and 22 live patients comparing the closest measured distances between tympanomastoid and posterior belly of the digastric muscle to the facial nerve. They proved that the tympanomastoid suture is a significantly closer and less variable anatomic landmark to the facial nerve than the posterior-superior margin of the posterior belly of the digastric muscle in parotid surgery.

The SMA enters the skull through the stylomastoid foramen, an orifice it shares with the egressing facial nerve. It seems logical therefore to use this relationship to aid the identification and preservation of the facial nerve during surgery.

Moreau et al. [7] anatomically dissected 30 facial nerves in fresh cadavers after arterial casting with red latex to provide specific information about the arterial-related anatomy of the trunk of the facial nerve from the stylomastoid foramen to its bifurcation. The trunk of the facial nerve was in proximity to the stylomastoid artery, which originated from the posterior auricular artery in 70% of the specimens, from the occipital artery in 20% and directly from the external carotid artery in 10%. The SMA passed medially to the trunk of the facial nerve in 63% of the specimens and laterally in 37%. The main shortcoming of this very good study was the anatomical nature of the dissection rather than the use of the standard surgical approach which would have allowed direct translation into clinical practice. Our results differ in that the SMA appeared to consistently pass lateral to the egress of the nerve in superficial to deep anterior to posterior surgical dissections. Some discrepancies can be accounted for by the fact of the differing nature of the dissections (anatomical versus surgical) with their significantly different head positions (anatomical rather than standard surgical).

The 8 cases in the clinical part of our study where the landmark was not useful are important in showing the difficulty in applying set approaches for any operative procedure. Due to the nature of the incision and dissection depending upon the varied pathology in some cases the surgical field was soiled with blood from the superficial dissection. This bleeding was not from the SMA but usually from superficial veins. In these cases using the SMA as a landmark was less helpful and other landmarks were used to find the nerve. As any other tool we do not propose this approach for all situations and circumstances only that it be in the surgeon's armamentarium to be employed when appropriate.

The data in this study supports the use of the SMA as a reliable landmark for the facial nerve which may potentially reduce morbidity. By the early identification of the SMA and by following it infromedially for a few millimetres we were able to consistently identify the location of the facial nerve. Identification aids in the preservation of the facial nerve by preventing inadvertent nerve section.

Since the SMA was found in only 80% of the clinical study this suggests that this artery may not always be present or identifiable. As radiologic studies are not always routinely performed, we used in order of personal preference (the tympanomastoid suture line, posterior belly of the digastric muscle, retrograde dissection of a peripheral branch of the facial nerve and tragal pointer). We also regularly use 'loupe' or formal microscopic magnification and a combination of nerve stimulator and nerve monitors. The surgeon needs all the help that can be manifest but nothing replaces clinical experience aided by anatomical dissection and helpful initial supervision.

## Conclusion

This anatomical aid, the stylomastoid artery, when supplemented by the other more commonly known anatomical landmarks and intra-operative facial nerve monitoring further reduces the risk of iatrogenic facial nerve damage and 'potentially' operative time. The drawbacks of this approach are that it requires early and meticulous attention to haemostasis combined with very gentle tissue handling. A feature that many traditional more approaches were not known for.

The surgeon should use as many of the available landmarks as feasible to perform safe facial nerve surgery. Both the main trunk and peripheral branches must be identified and preserved to prevent permanent aesthetic sequelae related to facial paralysis.

## Competing interests

The authors declare that they have no competing interests.

## Authors' contributions

TU, WJ, SARN, SUS designed the study, carried out the literature research, clinical and anatomic study and manuscript preparation. AS, HS, CH were responsible for critical revision of scientific content and manuscript preparation and review. All authors read and approved the final manuscript.

## Consent

Written informed consent was obtained from all of the patients for publication of these cases and any accompanying images.
